# Self-monitoring of health - user viewpoints on gathering data using consumer health technologies during leisure time

**DOI:** 10.3389/fdgth.2025.1588183

**Published:** 2025-06-30

**Authors:** Nora Weinberger, Martina F. Baumann, Maria Maia

**Affiliations:** Institute for Technology Assessment and Systems Analysis, Karlsruhe Institute of Technology, Karlsruhe, Germany

**Keywords:** consumer health technologies, wearables, brain-activity monitoring devices, digital health, user perspectives, technology ethics, technology assessment

## Abstract

**Introduction:**

Consumer Health Technologies (CHTs), including wearables and brain-activity monitoring devices, are increasingly integrated into everyday life, extending beyond clinical settings into leisure activities. Yet, their ethical and social implications, especially in unregulated, non-clinical contexts, remain underexplored.

**Methods:**

This qualitative study examines how individuals perceive and engage with CHTs by combining guided interviews and pre-interview questionnaires. It focuses on attitudes toward health data collection, data sharing, privacy concerns, and the use of EEG-supported devices.

**Results:**

Findings reveal a complex landscape of trust and concern. While participants generally favored sharing data with research institutions over corporations or insurers, they were skeptical about broad consent models. Some acknowledged potential health benefits of CHTs and EEG-supported technologies, but also expressed concerns about data security, behavioral pressure, and the normalization of self-optimization.

**Discussion:**

The results underscore the need to center user perspectives in the development of CHTs, to promote transparent and context-sensitive privacy policies, and to anticipate ethical implications. In particular, the article argues for ethical frameworks to guide the use of EEG-supported technologies in everyday settings, ensuring alignment with societal values and equitable access to digital health benefits.

## 1 Introduction

Advances in technology and science, as well as the capacity to collect, collate and analyze extensive datasets, are paving the way for new frontiers in health research and therapeutic advancement. Consequently, digital health technologies, including smartphone applications and wearables, collectively termed Consumer Health Technologies (CHTs)^1^, are now commonly integrated in citizens' daily leisure and work lives (1, 2). As an illustration, in Europe “*monitoring one's health has been an important topic for many even before the COVID-19 pandemic*” (translated by authors*)* (2, p. 2). These CHTs enable users to (pro)actively monitor health data, share it via social cloud services, and in certain instances, even interpret or analyze the data themselves (3, 4). The international Open Humans^2^ initiative, and the European Smart4Health^3^ project exemplify these developments, facilitating the secure management and sharing of citizens' health data across national borders. In Germany, for instance, CHTs can now even be prescribed by doctors and psychotherapists, with the costs covered by statutory health insurance^4^ (5, 6). The integration of CHTs into the healthcare system, coupled with their pervasive adoption, has also stimulated the use of citizen-generated health data in medical (research) studies, thereby facilitating a more comprehensive understanding of health behaviors and outcomes.

Despite the potential benefits of CHTs, including their use in prevention and diagnosis (7), as tools for clinical studies or health services research (8), and for optimizing therapies (9, 10), certain studies have identified possible adverse effects at various levels. These include psychosocial consequences, such as emotional distress, and a potential erosion of solidarity principles in health. This is corroborated, for example, by findings from studies conducted by (7, 11), and (12). These concerns about solidarity are particularly pertinent in Germany, given the unique structure of its health insurance system, which is based on solidarity-driven contributions and largely uniform benefits across socio-economic groups (13). Furthermore, additional challenges emerge with CHTs, such as concerns about the voluntary sharing of data for the development of data-based medical products, as well as questions about the (in)equitable involvement of citizens [cf. e.g., (14)], in order to gather their perspectives on the use of citizen-generated health data.

However, regardless of research efforts into these consequences, scholars still argue that the implications and risks associated with the collection and use of CHT data—particularly when conducted in private, unregulated settings (“leisure”) using commercial devices—remain under-explored in public debate and scholarly research (15–18). Moreover, research on the ethical and societal dimensions of CHTs remains based on relatively limited empirical evidence, although ethically sensitive issues in CHT data use include health insurance bonus schemes that incentivize CHT use (15), and the expansion of CHT applications to highly sensitive data categories. However, the emergence of technology developments, such as wearables with electroencephalography (EEG), has also raised previously unknown ethical concerns, particularly in countries with strict data protection regulations.^5^ In response to these persisting ethical challenges (12), call for a nuanced conceptualization of these potential drawbacks and meticulous, comprehensive examination of their implications.

Against this backdrop, this article presents findings from a study examining the ethical and social implications of leisure-generated data collection and data use of CHTs and, to date, basically uninvestigated EEG-based CHTs. Although the study addressed two primary areas, (i) the application of accumulated data in medical care and (ii) data collection for health promotion and prevention in leisure contexts, this article focuses exclusively on the latter [for details on the former, please see e.g., (11) and Baumann et al., submitted manuscript]. Therefore, our article examines the following questions: *(a) What risks and side effects do individuals associate with the use of CHTs in leisure contexts? (b) What is the citizen perspective on data privacy and the use of CHT data by CHT-companies*, for research, and health insurers? *(c) How do respondents evaluate health insurer incentives that promote healthy behaviors* via *CHT use? (d) How do respondents perceive EEG-supported CHTs for measuring and analyzing brain activity?*

Based on these research questions, after describing the methodological approach, this article provides insights into different social and ethical aspects of health data collection in leisure contexts from the user's perspective.

## 2 Methodology

### 2.1 Participant recruitment

To recruit participants, posters were displayed in various public spaces within the study region (a major city in Germany), such as the city center, shops, medical practices, pharmacies, museums, educational institutions, and sport associations. Additionally, an advertisement was published in a local newspaper, and calls for participants were shared via:

• Twitter (now: X) using relevant hashtags such as #digitalhealth #mobileapp #wearabletech #mobileapp #wearable #sensors #Daten ‘#data #Gesundheitsdaten #healthdata #BigData #quantifiedself #selftracking #optimizedself #selbstoptimierung #healthcare #selbstvermessung #Gesundheitsapps #healthapps #fitnessapps #lifelogging #Fitbit #Polar #Runtastic #Garmin #Apple #biofeedback #fitnesstracker #strava,
• Facebook groups (including Quantified Self Germany, Fitbit Germany, Fitness Tracking League, Fitness Tracker, Strava Runners, Garmin Fitnessgroup Germany, Garm*in vivo*active 3 & Vivoactive 4 Germany, POLAR V800, Vantage V, V2, Grit X % Co, and GARMIN Fenix, Forerunner Fans, Fitness Junkie),
• Open Humans and Smart4health forums.

Individuals aged 18 years and older who had used or were still using a CHT were eligible to participate. There were no additional inclusion criteria. Interested individuals were also encouraged to refer others who might be relevant to the research questions [a method known as snowball sampling; see e.g., (19)]. The recruitment strategy followed an open approach, with no predetermined sample size calculation. This is consistent with the logic of qualitative research, where the aim is not statistical representativeness, but the generation of in-depth, context-rich data. We therefore sought to include individuals who contribute rich, reflective perspectives on the core phenomena under investigation (see research questions above).

A total of 24 individuals expressed interest in participating in the study through the multiple recruitment channels described above. Due to the open recruitment strategy and the snowball sampling method used, the response rate could not be calculated [see, among others (20), on unknown total population sizes]. In the end, the sample consisted of 21 participants (see Table 1), as some individuals declined to participate due to time constraints shortly before their scheduled interviews. This demographic overview reflects a generally privileged, urban, and tech-savvy sample, which could influence the general applicability of the study's findings to broader, more diverse populations.

**Table 1:** Sample characteristics.

Outcome	N	%
Gender		
Female	13	62
Male	8	38
Diverse	0	
No answer	0	
Age (years)		
18–25	5	24
26–35	9	43
36–45	0	
46–55	3	14
56–65	3	14
No answer	1	5
Marital status		
Single	11	52
Married	10	48
No answer	0	
Education/graduation level^a^		
Hauptschule (graduation 5 years after elementary school)	0	
Realschule (graduation 6 years after elementary school)	2	9.5
Abitur (graduation 8–9 years after elementary school, university entrance qualification)	16	76
Others	2	9.5
No answer	1	5
Main occupation		
Gainfully employed and working	14	66.5
School pupil/student	5	24
Pensioners	2	9.5
Other	0	
No answer	0	
Population/Residence		
>100.000 (big city)	12	57
50.000–100.000	3	14.5
20.000–50.000	2	9.5
5.000–20.000	4	19
<5.000	0	
No answer	0	
Technical affinity		
Not tech-savvy	0	
Hardly tech-savvy	0	
Rather tech-savvy	7	33.5
Tech-savvy	6	28,5
Very tech-savvy	8	38

^a^The education/graduation level refers to the duration of secondary school following the four-year primary school.

### 2.2 Study design and data collection

To address the aim of the study, a primarily qualitative research with supporting quantitative elements was chosen [see for example (21, 22)]. This combined approach allowed us to contextualize participants' statements and guide interview prompts based on prior individual responses. Accordingly, the study followed a qualitative design that included a narrative literature review to inform the interview guide and the deductive coding framework, as well as qualitative modules that sought to elucidate the perspectives of CHT users. In addition, a short pre-interview questionnaire was used to collect data on usage routines and personal beliefs about data privacy. While the questionnaire data were analyzed descriptively, for instance by summarizing response frequencies, their primary purpose was to contextualize the interviews and support the development of individual prompts. Selected findings (e.g., participants' consent model preferences) are referenced in the article to support the qualitative analysis, but no independent quantitative analysis was conducted.

### 2.3 Measures

#### 2.3.1 Narrative literature review

To gain an initial overview of CHT use and its facets, a narrative literature review was conducted [for further details, see (11)]. This review served to contextualize the study within the current state of research and to inform the development of both the interview guide and the initial coding framework. Based on insights from the literature, a set of guiding questions was formulated. These questions were then subject to a process of evaluation, refinement and organization through multiple rounds of review by the project team in conjunction with external researchers, and subsequently adjusted based on a pre-test.

#### 2.3.2 Guided interviews

Following the granting of ethical approval, semi-structured individual interviews were conducted. Each interview lasted up to one hour and covered the following areas: (i) participants* use patterns and reasons, (ii) their expectations, (iii) their experiences with devices, apps, and services, and (iv) any concerns and perceived risks. Subsequently, questions were posed regarding (v) data use by third-parties, (vi) data privacy and informed consent, as well as (vii) current trends and debates, including an increased health-related responsibility and (rewarding of) health-conscious behavior (for example, through health insurance bonus programs). Finally, participants were invited to share (viii) their views on data collection through EEG-supported CHTs, using brain activity measurement as an illustrative example.

Prior to the interviews, participants were fully informed of the purpose, aims and content of the study, as well as details of data protection. They were given sufficient time to decide whether or not to engage on the study and were permitted to withdraw their consent at any time. Information sheets in accessible language were prepared and signed by each participant. Interviews were conducted online or in person at a location of the participant's choice, depending on their preference. Based on the overall impressions from both online and in-person interview, participants' responses and the level of involvement of the participants appeared consistent, indicating reliable data quality irrespective of chosen interview mode. The interviews were digitally recorded and transcribed anonymously by an external service provider in accordance with strict confidentiality and data protection requirements. In addition, memoranda were prepared which provided a brief account of the content of the interviews and a description of the interview situation.

#### 2.3.3 Questionnaire

To complement the qualitative interviews, a questionnaire of 15 closed ended questions was sent to each participant several weeks before the scheduled interview. This was done to ensure that relevant findings could be referred to during the interview, and to combine qualitative with quantitative results. The survey focused on the participants' use of CHTs, including the technologies used and the duration of use. Sociodemographic information (e.g., gender, age, marital status, education, occupation) was also collected. In addition, the questionnaire addresses technology affinity and privacy concerns, e.g. “Do you think about what happens to your data from CHTs? Have you read the privacy policy of your CHT?” (translated by the authors). Besides of this, participants were asked about aspects of informed consent. For this purpose, they were presented with an explanatory text, after which they had the opportunity to select one of three consent models:

Modell A: Broad Consent – You allow researchers to use all CHT data for any research purpose, including future studies not explicitly described at the time of consent.
Modell B: Study-Specific Consent – You give consent solely for a single study, with a clearly delineated scope and specific data usage.
Modell C: Dynamic Consent - This model allows you to set individual preferences for data sharing, including the option of limiting data usage to certain purposes. You can adjust these preferences at any time, thereby conferring upon this model its “dynamic” nature.

In addition, participants were introduced to a CHT data management application, which provides options to (i) release only specific CHT data for selected research purposes (ii) receive updates and results of studies using their data, and (iii) access general information on ethical and legal issues, such as data privacy, and contact details of ethics advisors and researchers. While the questionnaire data were summarized descriptively, for example using frequency counts, no separate quantitative analysis was conducted. Selected results, such as consent model preferences, are referenced in the results section to illustrate specific participant viewpoints.

#### 2.3.4 Data analysis

For the qualitative content analysis [in line with methods outlined by (23–25)], all team members undertook multiple readings of the interview transcripts. This approach was taken to ensure a comprehensive understanding of the material, in line with the aims of the study. The transcripts were then intensively and iteratively discussed to enhance understanding and to identify patterns within the data. Subsequently, the transcripts were examined word-for-word, and reflections and observations (e.g., similarities and differences among participants) were duly documented. First, the transcripts were analyzed *deductively* by coding relevant segments into predefined categories (26). These categories were theory-driven in their selection “*that means, specifically based on characteristics that, according to theory and the current state of research, appear important to the research question*” (27, p. 7).

The primary categories were derived from the structured interview guide. Each researcher developed an initial coding scheme which was then discussed, merged, discussed again and redefined by team consensus. To strengthen the interpretative validity of the coding categories, this process combined initial independent coding with iterative joint review. The team comprised researchers from the fields of technology assessment, medical ethics, and science and technology studies, aged between 30 and 50 years, all with prior experience in qualitative research on digital health technologies. This disciplinary and experiential diversity supported analytical transparency and thematic sensitivity. The final coding scheme was reviewed by one team member (MM) and adjustments were made to categories and subcategories as necessary. This process resulted in 25 main categories and their respective subcategories. These reflect the core topics of the interview guide as well as recurrent themes in the empirical material (see Table 2). For better readability, code names have been translated and formatted using conventional spacing and capitalization.

**Table 2:** Final set of categories and sub codes used in the qualitative content analysis of interview data on Consumer Health Technology use. Not all categories were further subdivided into sub codes, as the empirical material did not warrant additional differentiation in these cases. These categories without sub codes are indicated by a dash (—) in the sub code column.

	Category (main code)	Sub category (Sub code)
1.	Reasons for purchasing the CHT	Control/monitoring; Sports activity; Reward; Tool for data tracking; Interest in technology; Usability; Interest in data and functions; Self-motivation; Fun; Health awareness; No conscious decision; Recommendation; Inexpensive/second-hand
2.	Reasons for changes in usage behaviour	—
3.	Interest in other data sources	Sleep tracking; Calorie consumption; Distance; Time; Heart rate measurement; Altitude meters; Body composition; Activities; VO2max; Fluid intake; Female cycle
4.	Goals associated with CHT use	Sport- and training-specific goals; Health-related goals; Self-tracking; Optimization; Motivation enhancement; No specific goal
5.	Expectations regarding the use of CHT	Unclear; Fulfilled; Not fulfilled; Luxury product
6.	Device change to meet expectations	—
7.	Behavioural changes resulting from CHT use	—
8.	Insights gained about oneself	Improvement through training; Recorded values; Fun using the device; Feedback/affirmation; Improved self-perception
9.	Personal use of the collected data	Data analysis practices; Data sharing; Motivation; Training control; Shared with physician
10.	Comparison of data with others	Friends; Family; Face-to-face; Messenger; Community/forums; No comparison
11.	Discussion of data with physicians/doctors	No discussion
12.	Perceived significance of data (for achieving personal goals)	High significance; Low significance; Realistic interpretation of data necessary; Unclear significance; Supporting tool; Change in significance over time
13.	Impact of COVID-19 on CHT usage behaviour	Increased use; Constant use; Decreased use; COVID as trigger for CHT use; CHT reflects immobility; Unclear
14.	Concerns and perceived risks regarding CHT use	Individual-level concerns and risks; Data-related concerns and risks; No concerns
15.	Assessment of data privacy with the device in use	Own data protection behaviour; Effort involved; Lack of transparency; Low level of data protection; Coercive situation; Depends on the company
16.	Reasons for concerns regarding potential data use	Data protection
17.	Reasons for little or no concern regarding potential data use	Lack of engagement with the issue; Risk–benefit ratio; Trust in companies; Data not perceived as sensitive; Own data protection behaviour; Unclear what happens with data; No concerns
18.	Wishes regarding the handling and use of CHT data	—
19.	Sensitivity to privacy protection Facebook/Instagram in comparison to CHTs	Depends on own usage behaviour; Higher privacy sensitivity on social media; Both perceived as private; Unclear
20.	Perceived responsibility for one’s own health	—
21.	Openness to using the two EEG technologies	Openness; No openness; Lack of information on the technology; Effectiveness; Change in attitude over time
22.	Concerns about the use of future technologies	Stimulation and measurement of the brain; Implementation and use of the technology; Performance enhancement; Negative health effects; Invasion of privacy; Benefit-effort ratio; Data protection; No concerns
23.	Willingness to share EEG technology data with research	More likely to share CHT data; Support for research; Depends on research institution and ethics; Depends on EEG technology; More likely to share EEG data; Depends on stage of development; Prefer private EEG use for now; No difference
24.	Expectations and recommendations for developers of future technologies	Usability; Clear development guidelines; Better data collection and quality; Consumer involvement; Data protection; Disease-specific/ health-specific; Affordable technologies; Free access to new technologies; Education about risks; Long duration of use; Support for small companies
25.	Concluding remarks	Interest in results; Questions about participant recruitment; Questions about the study; Suggestions

The relevant quotations were then organized into the respective categories and subcategories [see e.g., (28), for this approach]. Subsequently, thematic units from participants' statements were grouped by meaning and abstracted into sub-themes, allowing three additional categories to be *inductively* derived from the empirical material (26): *CHTUsed*, *DurationofCHTUse* and *SocialMediaUsed* (translated by authors). Statements from the interview were also assigned to the additional categories [e.g., (29)]. While this coding framework included 25 main categories and several sub-categories, the present article focuses on a selected subset directly related to our guiding research questions, namely, participants' views on benefits and risks of CHT use, data privacy and third-party data sharing, consent models, and EEG-supported technologies.

The reliability and validity of these interpretations were strengthened by repeated independent and joint readings of the empirical material by researchers with diverse disciplinary backgrounds and substantial thematic expertise. In addition, the multiple reading was “*the basis for thoughtful and rigorous discussions that were grounded deeply in the text and focused on creating the best possible account of meaning of the data*” (30, p. 387). The final data analysis—assigning quotes to deductively and inductively generated categories—was performed using MAXQDA^6^ software.

#### 2.3.5 Ethical approval

The study was conducted in accordance with the Declaration of Helsinki. In addition, the interviews and the survey were approved by the Ethics Committee of the Karlsruhe Institute of Technology on 1 October 2021. After receiving the informed consent the respondents gave their written consent to be reviewed and signed before the interviews began.

## 3 Results

The following section presents the study's results, with focus on key issues central to the research questions of the article (see Introduction). We do not delve into participants' general usage patterns and motivations for engaging with CHTs, nor do we report all coded categories from the full analytical framework. Only those categories that directly address our research questions are presented here, namely risks and side effects, data use by third-parties, data privacy, and EEG-supported CHTs. [see (11)]. Additionally, participants' experiences with devices, apps, and services are included only insofar as they relate specifically to these focal topics.

### 3.1 Perceived benefits and Side effects

When asked whether CHT use had influenced their behavior, participants reported a range of effects. Some stated that their motivation for physical activity had increased and that the devices had spurred them on to make improvements, as one participant explained: “*Yes, I’m definitely more motivated to run and always try to improve times*” [cht01_w_18-25]^7^. Others remarked that although the CHTs had positively influenced their activity levels, they believed they would still lead a health-conscious lifestyle without them, as one young participant explained: “*I’d say that the app does make me do more sports through tracking, but […] even without the watch, I’d live a healthy and mindful life*” [cht01_w_18-25]. In contrast, some interviewees expressed the view that CHTs constituted a significant source of stress and placed them in a position where their motivation was in conflict with the pressure to improve, with one participant describing it as follows: „*But sometimes it's a shame when the focus shifts from ‘I’m going for a run' […] to ‘I have to be faster, I have to get better’*“ [cht01_w_18-25]. And another participant: „*I think, it's more negative. At times, it was really more like living by the watch*” [cht18_w_18-25].

The ‚urge to get better and better‘ was also mentioned when discussion concerns and risks associated with the CHT use. A younger participant, for example, now misses ‘the “*fun of running*” due to the internal pressure to “*improve performance*” [cht01_w_18-25]. Another participant saw a risk that “*one could overdo sports*” [cht11_w_26-35]. Alongside the strain of aiming to meet certain performance targets, some respondents expressed fears about a trend toward normalization, particularly among young people that are unconditionally orientated towards any data as a normative standard. In general, in their opinion, society is already strongly shaped by certain “*norms about how people should be*” [cht03_w_46-55], which are further reinforced by CHTs and the constant availability of data. According to the participants, another risk could also be the “*false sense of security conveyed by data, which poses a serious issue, especially for people with pre-existing conditions*” [cht04_w_26-35]. Some users also emphasized risks associated with data privacy, including the potential sharing of sensitive data with third parties and the misuse of their information (see also sections above). Furthermore, several participants voiced concerns regarding possible surveillance. One participant expressed this sentiment as follows: “*So I would see it very critically if people could be ‘observed' through this. Right? I mean, it doesn't have to be an official rule, but of course you can also monitor people using financial incentives. […] That's why I wouldn't share the data with my health insurance company, even if they gave me a financial bonus for it […] that’s going too far for me.*” [cht10_m_26-35].

### 3.2 Data sharing for research and consent models

In terms of their potential willingness to provide data for research purposes, the majority of respondents indicated a general willingness to share their data for health research in the survey. However, their willingness was highly dependent on the specific research purpose (20 out of 21 participants). For eight participants, however, it was not only the purpose but also the data protection regulations that were important (note: multiple answers were possible). Six of the 21 respondents indicated that they would provide their data unconditionally, while only one respondent indicated that he would not provide this data for research purposes at all. The interview results also indicate that participants were generally willing to provide data for academic research and clinical trials, as long as “*no mischief is done with it*” [cht03_w_46-55]. In contrast, respondents were more cautious to very skeptical about the commercial use of CHT data by companies or health insurance companies, and all participants opposed the use of CHT data for military purposes.

In addition, participants' consent to share health data depended strongly on the intended use: while there were few objections in the survey to using data for drug development and basic research, comparisons of health status across (and between) different groups, such as ethnic or socio-economic groups, were particularly controversial. Ethical and data protection concerns were raised, as well as opposition to, for example, the “*commodification of health data, such as adjusting health insurance premiums based on how healthy someone lives*,” “*targeted advertising development*,” or research “*without significant benefit to the public*”.

The surveys also showed that receiving information about study results was very important (13 out of 21participants) or at least important (eight participants) after participating in a research study to which they had contributed data. Additionally, the interviews revealed that, for some participants, general awareness of the value of data in research was only partially developed: “*On the other hand, what can one really do with the data?”* [cht03_w_46-55].

With regard to the preferred consent models, the findings indicate that ten participants would choose Model B “Study-Specific Consent,” seven participants preferred Model C “Dynamic Consent,” and six participants would opt for Model A “Broad Consent.” The study also showed that most participants would likely use a CHT data management app (very likely: 8; somewhat likely: 8). It was important to participants to emphasize that data should not be shared through this app; rather, the app should only determine who has access to their data. Three participants were unsure if they would use the app, and the remaining participants were less inclined to do so (somewhat unlikely: 5; very unlikely: 2).

### 3.3 Data privacy awareness and concerns

Responses to questions on data privacy revealed relatively consistent views and approaches among participants. The results show that all respondents considered data privacy to be relevant, yet all but one admitted to either not reading or only briefly scanning the privacy information (20 out of 21 participants). This was partly due to a lack of interest and a level of trust in the companies, as one participant illustrated: “*[…] So, I have no idea which servers the data gets transferred to or how secure the servers are. To be honest, I’m not super interested in that either. And maybe there's some trust in it that makes me think: ‘Oh, it's such a big company, they have good rules, they’ll stick to them, and it’ll be probably pretty secure*” [cht10_m_26-35]. Another participant added, “*I assume they comply with data protection in accordance with European standards. Therefore, I believe that we’re protected. But nobody really knows*” [cht21_m_56-65].

On the other hand, participants noted that the privacy information itself was often “*not user-friendly*” [cht03_w_46-55] and “*too lengthy, so it doesn*’*t get read*” [cht17_w_single_18-25]. As one participant commented, “*Who reads a five-page privacy policy written in legal jargon? I think you could convey this much better in just two or three clear, concise sentences*” [cht10_m_26-35]. Another participant compared it to the terms and conditions, where people simply check the box without reading: “*It's just window-dressing from the data protection authorities to say people need to be informed. That's not real clarification for me; everyone just clicks it away*” [cht06_m_56-65]. Only one participant mentioned reading the privacy information thoroughly, though she added, “*Well, it reads well, that data protection is guaranteed in any case. But, of course, you never know whether that's true*” [cht20_w_46-55].

Additionally, some respondents contextualized data protection and data use within a broader framework. As one participant put it: “*[…] big companies, whether it's Google or others, regardless of whether I have a smartwatch or not, they already know way more about me than what they could get from my watch. […] It starts with something as simple as using a navigation system*” [cht06_m_56-65]. Other participants summarized it as: “*At the end of the day, we always say, yeah, they already have all our data anyway, so my watch data doesn*’*t make a difference anymore*” [cht22_w_26-35], and, “*But I honestly believe that's utopian these days anyway. Unless you don*’*t use the internet, Google or Facebook products or anything else. Somewhere, somehow, there's always a data record about me or someone*” [cht23_w_18-25]. According to participants, many people do not even consider the data they are sharing, as the benefits of CHTs and other technologies often outweigh privacy concerns.

The interviews further indicated that participants viewed the reuse of data by CHT companies critically. One participant expressed this sentiment well: “*What bothers me is that I would like to use the app without the data being uploaded to [company]. I’d like all my data to stay with me, and I could still use the full functionality. Unfortunately, with [company], that's not possible. So, if I want to use the full range of features in the app, I have to share my data with [company]”* [cht11_w_26-35]. Many companies also do not make it clear what happens to the data or how it is further used, as two participants noted: “*But what [company] ultimately does with my data, I don*’*t know 100%*” [cht16_m_26-35] and “*Honestly, I don*’*t know what they do with the data*” [cht19_m_18-25]. This concern extended beyond health data to other data types, such as location and the linking of different data sources. In participants' view, “*as little user data as possible should be uploaded to the [company's] servers […] and all user data should be kept on the user's device whenever possible*” [cht11_w_26-35]. One participant summarizes this as an indication to the technology developers as follows: “*Also, data privacy options should be clearer. I think it's very important to be able to share your data […]. Yes, it should be absolutely clear how to do it, where it's stored, and above all, the interface should be more user-friendly. So, that would really be my advice to the producers of health technology because it's actually a disaster right now*” [cht04_w_26-35].

But, overall, the interviews showed that many respondents saw potential benefits in data reuse, as long as the user himself decides “*who gets access and when*” [cht08_m_26-35] and the data is used only for the purpose to which they have “*explicitly*” consented [cht12_m_46-55]. Among the benefits, respondents stated a stronger integration of CHT data into the healthcare system, with the United States mentioned as an example: “*And in the U.S., as far as I know, these services are much more integrated into the healthcare system. So there, I can actually go to the doctor and say: “Look, I’ll print out my ECGs from my watch for the last six months’ or something like that*” [cht08_m_26-35].

### 3.4 Responsibility for personal health

In addition to questions regarding usage patterns, data reuse, and privacy, the interviews addressed the growing expectation for individuals to assume responsibility for their own health. For example, some health insurers offer premium discounts to policyholders who cease smoking or participate in activity programs, such as the Generali Vitality Program^8^ (see also the Background). The findings reflect a range of perspectives on this development. A large number of participants viewed it as a positive trend, believing it could reduce costs and foster self-responsibility and motivation, given that “*many people, especially in industrialized nations, are significantly less active and eat too much*” [cht06_m_56-65]. Another participant expressed a similar view: “*I think it's good in principle; I can*’*t sit around my whole life and then shout for help and say: “Yes, now others have to bear the costs of my illness.’ I think that's a very good thing, and I believe anyone who, let's say, is willing to eat a little healthier, to exercise, is really on the right track*” [cht20_w_46-55]. One respondent mentioned that he personally participates in health programs and share his step data with his health insurer.

Participants also believe that this trend could lead to a greater awareness of collective responsibility and solidarity. In many cases, interviewees referred to the COVID-19 pandemic to illustrate this point. As one participant described: “*The Corona debate is also about solidarity with other groups of people […] it's also about this sense of responsibility, for example, that my actions, so to speak, also affect the lives of others. When it comes to infection chains […], I firmly believe that we all bear responsibility, as adults, and should bear it, not only for ourselves but for society*” [cht03_w_46-55].

Overall, interviewees cautioned that such programs should be based on individual targets and implemented with clear limits, emphasizing that these programs should remain incentives rather than “*mandatory or semi-mandatory”* [cht23_w_18-25]. Otherwise, there could be growing pressure on those who choose not to participate in bonus programs or preventive offerings, such as those who “*simply don*’*t want to exercise*” [cht04_w_26-35] or who “*want to eat sugar*” [cht06_m_56-65]. Ultimately, this could—in participants’ view—lead to misuse of CHTs (e.g., faking step counts, giving the device to someone else) and, in the worst case, even to the social “*exclusion of individuals*” [cht04_w_26-35], which would contradict the principle of solidarity. Participants noted that the long-term risks of such bonus programs are still uncertain. They also pointed out that health cannot always be influenced by health-conscious behavior alone, and that social factors, such as poverty, are often overlooked in this debate. For these reasons, some participants preferred alternative approaches and tools to promote health-conscious behavior. Nevertheless, some participants found it acceptable for health-promoting (i.e., “*reasonable”*) behavior could lead to lower premiums and additional benefits, while unhealthy behavior could lead to higher premiums. However, a distinction was made for people with illnesses; for example, one participant argued that diabetics with poor blood glucose levels should not have to pay more for the insurance than diabetics with better control. Additionally, some participants raised concerns about how health insurers handle health data (see previous sections on data privacy).

### 3.5 Openness and willingness to Use EEG-supported CHTs

Another part of the interviews and questionnaires focused on attitudes towards advanced future wearables, particularly technologies that measure EEG signals—such as brain activity to monitor attention levels during leisure time: devices could stimulate attention through a headband or detect and filter out noises deemed disruptive. A number of participants expressed initial openness and curiosity about trying out such EEG-supported CHTs, especially if they would improve their “*own situation*” [cht12_m_45-55] or enhance productivity at work or school. However, participants were uncertain about long-term use, as one younger participant put it: “*But actually using it regularly?”* [cht17_w_18-25]. At the same time, many participants emphasized their fears about the potential for continuous surveillance, possible perceptual influences and unintended behavioral changes. One participant shared his perspective as follows: “*If there's a technology that sustainably helps me work better and stay more focused, then I’d definitely like it—as long as there are no side effects. But if this technology is used to make students, for instance, in some kind of these learning factories, ultra-focused all the time, and if they look away even once, they get an electric shock to the back of their head or somethings, then I’m definitely not okay with that. So, it's crucial to differentiate exactly how, yes, how it's designed. You also have to consider whether phases of distraction or lack of focus serve a purpose, like letting the mind relax, or perhaps it subconsciously stores and collects information during those times*” [cht07_m_26-35]. In general, another participant noted that the “*threshold between taking advantage and risk and being beneficial for me, i.e., for my own wellbeing, […] is damn thin*” [cht21_m_56-65]. Two other participants raised anxieties about using EEG-supported CHTs that monitors attention *levels: “[…] and then, if you stop using the device, you might feel even worse or something because maybe you’ve been pushing yourself past your energy limit” [cht11_w_26-35] and “[…] maybe without the device, you might not be able to perform as well, since you’re no longer receiving that extra stimulation or optimization*” [cht19_m_18-25].

Some participants questioned whether, for example, noise filtering technology that works on the basis of EEG signals would be universally beneficial. One participant referenced the importance of sounds like police sirens and alarms, which some might find disruptive, even though “*it's a noise, yes, everyone should hear. So, I think that would be one of the biggest questions I’d have for this system. Yes? How does the system determine what bothers me and, and what should be filtered out in first place*?” [cht08_m_26-35]. Some participants made the potential use dependent on whether such a system could be worn externally, like a watch, or would need to be implanted to function effectively. They also emphasized that the use of these devices should not be mandated by employers or other authorities: “*As long as it's all voluntary and you can decide for yourself when to use it, then it's definitely an option*” [cht20_w_46-55].

In addition, privacy and third-party access to data was a major apprehension for participants, who stressed the need for strong and transparent regulation. They felt that brain activity data “*should stay with me and not, for example, be shared with my health insurer*” [cht15_w_26-35]^9^. They called for “*a system as closed as possible*” [cht15_w_26-35]. Nevertheless, some participants were amenable to providing EEG data for research purposes, as long as the use of the data is limited to a specific purpose, the research is conducted independently. They emphasized the importance of adhering to a clear ethical framework and ensuring transparent communication about the research goals and the intended data use. In contrast, a second group of participants could not envision using these EEG-supported CHTs at all or remained highly skeptical, noting, for example, that “*tracking brain activity is different from tracking pulse*” [cht01_w_18-25]. They also felt there is no personal benefit or “*added value”* in using such a device [cht11_w_26-35]. One participant explained: “*At first glance, I’d say it's going a bit too far for me. […] I don*’*t currently see any value in tracking*” [cht01_w_18-25], while another stated, “*I don*’*t think I need it because I don*’*t mind, for example, sitting in a noisy restaurant*” [cht09_w_26-35]. Participants also expressed concerns about continuous human “*optimization*,” which they feared could lead to “*side effects”* [cht07_m_26-35], such as psychological issues. As a result, one participant argued for age restrictions on the use of such cognitive CHTs. Other participants drew a distinction between different EEG technologies, suggesting that they might be open to the use of EEG-based tools depending on the specific functions enabled by EEG data.

## 4 Discussion

### 4.1 Participant demographics and characteristics

As shown in previous studies [cf. e.g., (31–34)], individuals with higher socio-economic status, especially those with higher levels of education, are more likely to use CHTs. This trend is reflected in the research sample, in which a majority of participants reported university-level education. In addition, the assessment of technology affinity showed that participants generally had a positive attitude towards technology. Rather than being a methodological limitation, this composition aligns with the profile of early adopters commonly observed in digital health research. However, it is important to note that the findings predominantly reflect the perspectives of individuals who are already familiar and comfortable with CHTs. Accordingly, the results should be interpreted as insights into the attitudes and concerns of experienced or interested users, rather than as representative of the broader population*.*

### 4.2 CHT benefits and Side effects

Participants' experiences with CHTs revealed both perceived benefits and unintended consequences. While some users associated the technologies with increased physical activity, enhanced motivation, reminders, enjoyment, validation, and improved self-awareness, these effects were not sustained across all cases. Several individuals reported that initial enthusiasm declined over time, often due to device inaccuracies, unmet expectations, or a growing disconnect between recorded data and subjective experience [see on this aspect, for example (35)]. These issues often resulted in frustration or a perceived tension between intrinsic motivation and externally triggered pressure to improve. Psychosocial side-effects such as stress, negative self-image and guilt were reported, as shown in other studies [see e.g., (36–38)], which not only affected satisfaction but also potentially hindered sustained use. In addition, aspects of technical usability and data quality contributed to a decline in motivation and long-term engagement with CHTs [see, among others (39–41),].

Usage patterns in the research sample were dynamic rather than stable. Participants adapted their engagement in response to shifting priorities, emotional responses to data, and other contextual factors such as life circumstances or personal goals. Over time, the novelty of the devices tended to fade, leading to a more selective or reduced use. At the same time, gamification features were experienced ambivalently, in some cases even by the same individuals, who described them as both motivating and pressure-inducing. While such mechanisms initially provided structure and incentives, they could also undermine users' sense of autonomy or reinforce performance expectations. These differentiated experiences underline how technical design features interact with users' health orientations and lived context [cf (39–41)].

These experiential dynamics do not merely reflect individual usage preferences but also point to broader ethical questions [see (11) for a more detailed discussion]. For instance, the reduction or discontinuation of CHT use was often not simply a matter of disinterest, but a response to perceived mismatches between user needs and system feedback [e.g., (41–43)], which may indicate a failure of inclusive or responsible design. Besides technical limitations, participants' reflections highlighted normative concerns about emotional and cognitive overload, and about the implicit moral assumptions embedded in CHT use. As discussed in earlier work [see (11)], the normalization of self-optimization in digital health can contribute to subtle forms of pressure, especially for more vulnerable users. At the same time, users were not passive recipients of these effects. Many developed pragmatic coping strategies, such as ignoring “frustrating” data, suspending use, or stepping back after an initial intensive phase. These responses can be interpreted as attempts to maintain autonomy and psychological balance in the face of technologies that promise control but may simultaneously impose subtle forms of behavioral governance.

### 4.3 Data privacy concerns and third-party data use

Participants consistently regarded data privacy as important, but admitted to reading privacy policies only briefly. This behavior was often attributed to a combination of trust in established companies and the complexity and inaccessibility of data protection documents [see on this aspect e.g., (44)]. Concerns about compliance with data protection regulations were especially pronounced regarding international companies, with some participants highlighting the need to monitor compliance with the European General Data Protection Regulation (GDPR), particularly in relation to US-based firms.

When reflection on the sharing of CHT data by third parties, participants expressed preferences and expectations depending on the intended data recipient. These differences were shaped by trust, perceived risks, and the anticipated benefits of sharing associated with various stakeholders, such as CHT companies, research organizations, and health insurers.

Many participants saw the sharing of personal data with CHT companies as a necessary trade-off: in exchange for app functionality, they tolerated sharing their data and limited transparency about how their data would be used. While some described this pragmatically, citing a “nothing to hide” mentality, others were more critical, particularly when it came to the handling of sensitive health data. This distinction between perceived data sensitivity and actual usage mirrored findings from previous studies [cf (44, 46)]. A recurring concern was the lack of control over how and by whom data would be used. Participants expressed a desire to use CHTs without having to share data by default, especially in cases where data handling practices remained unclear. This contributed to a growing skepticism towards commercial providers. As highlighted in in earlier research [cf (47)], the ability to control data access was often regarded more important than mere transparency. While many participants were open to sharing data under specific conditions, such as clear purpose specification and user control, they also called for mechanisms that would allow them to restrict data access and prevent secondary use. A minority even argued that if companies commercially exploit user data, including data generated through CHTs, users should receive financial compensation, an argument that resonates with ongoing debates about data donation models and digital reciprocity (48).

In contrast, data sharing for research purposes was generally viewed more favorably, especially when the research served public health aims and was conducted by trusted institutions [cf (49–51)]. Participants tended to focus less on the nature of data and more on who would use it and for what purpose, an attitude mirrored in other studies [e.g., (45, 52)]. Consequently, participants did not differentiate between the sharing of basic data, such as step counts collected through CHTs, and more complex data, such as EEG readings from advanced wearables. Furthermore, as shown in a study by (44), it was essential for participants that the intended purpose of data use be communicated clearly, using accessible language. Feedback mechanisms were also relevant: all participants wanted to be informed about research outcomes to which they had contributed data [e.g., (53)], and some viewed such ongoing updates as a prerequisite for trust. These preferences align with findings from studies on transparency in data-driven public health tool (52).

The idea of sharing data with health insurers provoked strong reservations. Participants expressed strong apprehensions that such data could be used to restrict benefits, increase premiums, or indirectly penalize individuals based on lifestyle or pre-existing health conditions—a concern also highlighted by (46). Additionally, several respondents explicitly questioned the compatibility of such practices with the solidarity principles that underline the German healthcare system. Even financial incentives were largely rejected if they were tied to data provision. These attitudes were supported by experimental research, showing that while financial incentives had minimal impact, trust and perceived risk significantly influenced willingness to share data [cf (54)].

In the context of international studies, our findings are consistent with broader trends in data sharing preferences. For example, participants in our study showed a clear reluctance to share data with insurers, but were more open to sharing with healthcare providers or researchers under certain conditions. Similar dynamics emerge in other studies: a study of older CHT users found a greater willingness to share data with physicians than with insurers or researchers (55), and a US study reported a significant willingness to share data specifically with healthcare providers (56). This reinforces the importance of context-sensitive data policies that reflect public expectations for privacy and trustworthiness. Moreover, our findings align with studies showing that societal risks, such as discrimination based on narrow or behavioral definitions of health, significantly impact willingness to share data with insurers [see e.g., (57)].

Overall, participants' willingness to share data was shaped less by the nature of the data itself than by the perceived trustworthiness of the recipient and the clarity of the intended purpose. While openness toward research institutions was generally high, scepticism toward insurers and commercial actors was widespread. These patterns underscore a broader ethical insight: data sharing in health contexts is not simply a matter of consent, but of perceived fairness, control, and alignment with normative expectations. Transparent and purpose-specific data policies that acknowledge these concerns are essential to ensuring the social legitimacy of data-driven health systems.

### 4.4 Preferred consent models

Participants expressed distinct preferences regarding consent models for data use. Only a third supported the concept of broad consent, which allows data to be used for unspecified future purposes. The majority preferred more granular approaches that offer transparency and user control. Most notably, study-specific consent, in which permission is granted for a single, clearly defined study, was the most frequently endorsed model. Dynamic consent, which enables users to adjust their preferences over time and specify acceptable data uses, was also viewed positively by several participants. These preferences align with findings from other studies [e.g., (49)], which indicate that broad consent is generally regarded as insufficiently informative and too open-ended.

These findings echo recent debates in bioethics that problematize the vagueness and lack of agency associated with broad consent models, especially in dynamic, data-intensive research environments. Scholars have argued that broad consent often fails to provide participants with meaningful control or adequate information about potential downstream uses of their data, which can raise concerns about autonomy and legitimacy [cf (49, 50)]. In contrast, study-specific and dynamic consent frameworks are increasingly promoted as ethically preferable options that respect individual decision-making and enable more contextualized forms of participation.

In addition, many participants indicated openness to using a CHT data management app that would support consent administration. However, they emphasized that such an app should function strictly as a tool for setting and managing permissions, without automatically facilitating data transmission or external access.

### 4.5 Acceptance of health insurance bonus programs

The empirical study also examined participants' views on the trend toward individual accountability for health-conscious behaviors, such as avoiding smoking and adopting healthier lifestyles. For example, health insurers incentivize their clients with premium discounts, encouraging them to track certain health parameters and use specific apps [cf (46, 58, 59)]. Approximately one-third of participants viewed this development positively, believing it promotes self-responsibility and motivation. They noted, in line for example with (58) that many people in industrialized nations tend to lead sedentary lifestyles and overeat, which could be positively impacted through such programs. One participant, for instance, mentioned actively participating in health programs and sharing step-count data with their health insurer. Others emphasized that such programs could foster greater responsibility and solidarity, reminiscent of the collective spirit observed during the COVID-19 pandemic [cf (60–62), for a selection].

At the same time, most participants considered bonus programs a suboptimal tool for enforcing responsibility, expressing concerns that such measures might contribute to an unsupportive insurance system. All interviewees stressed that bonus schemes should operate on an individual basis (e.g., tailor-made targets) with clearly defined boundaries. They underlined that these programs should provide incentives without becoming mandatory or semi-mandatory in order to avoid creating pressure or coercion. Some participants warned that making such programs obligatory could encourage misuse of CHTs, for example falsifying step counts or allowing others to use the device. These behaviors, also noted by (63), could lead to social exclusion and contradict the principle of solidarity.

In addition, participants highlighted that health is not always influenced by individual lifestyle choices. They referred to structural and social determinants such as poverty, which are often overlooked in the design of such incentive systems, a critique echoed in the literature [cf (64)]. These concerns reflect broader debates about data justice, which emphasize the need to ensure that data-driven health systems do not reinforce existing inequalities or shift responsibility for health outcomes solely onto individuals. From a data justice perspective, incentive schemes based on behavioral tracking can risk amplifying disadvantage if they overlook the socio-economic conditions that shape health behaviors and data access. Several participants implicitly echoed such concerns, pointing to the risk that bonus programs could undermine solidarity and fairness within the healthcare system. While others found it acceptable that health-promoting behaviors might lead to reduced premiums or be rewarded with additional services, they clearly differentiated such cases from those involving individuals with medical conditions. For example, people with diabetes should not face higher costs solely based on blood sugar levels.

Finally, participants expressed concerns about how health insurers handle health data in the context of such programs and called for clear privacy regulations (65–68) found that privacy concerns strongly influence attitudes towards pay-as-you-live (PAYL) insurance models.

### 4.6 Ethical challenges of EEG-supported CHTs

Participants' views on advanced CHTs that use EEG signals to monitor brain activity and attention or filter noise reflect a mix of curiosity, caution and ethical concern. While some participants initially expressed interest in EEG-based devices, especially for health improvement and productivity, this openness declined when participants considered long-term use. Concerns were raised about potential side effects, such as unintended behavioral changes, continuous surveillance or perceptual manipulation, as noted similarly in a study by (69).

Participants emphasized the need for careful design to avoid such unintended consequences. They argued that while technologies that enhance concentration might be beneficial in some contexts, moments of distraction also serve important cognitive and emotional functions, in line with perspectives on the balanced functionality of attention regulation [cf (66)]. Accordingly, many described the line between benefits and risks of EEG-supported CHTs as narrow [cf. e.g., (70)]. Some feared that stopping the use of such devices could reduce performance and well-being, suggesting a risk of psychological dependency. These concerns parallel general CHT usage patterns, where emotional attachment to data or loss of autonomy can arise [e.g., (71)]. For example, one study has shown that step counters can increase physical activity but simultaneously decrease enjoyment of walking [see e.g., (72)]. Participants also expressed scepticism towards noise-filtering features, especially when they might suppress important environmental cues, such as sirens or alarms.

Acceptance of EEG-based CHTs appeared to depend strongly on context and purpose. Participants showed greater openness to EEG-supported CHTs when they address specific health needs, for instance, in the treatment of conditions like Attention Deficit Hyperactivity Disorder (ADHD), rather than general This aligns with studies on neurofeedback, which show higher acceptance when EEG-based interventions are seen as alternatives to conventional therapies [e.g., (73)]. However, several participants questioned whether such applications could transfer meaningfully into everyday life. Factors such as whether the technology is externally worn or implanted, and whether its use is voluntary, played a significant role in shaping perceived legitimacy. Privacy and third-party use also emerged as key concerns, echoing previous research [cf (74)]. While some participants were willing to share EEG data for research, provided ethical standards were met and data use remained transparent, others viewed brain activity tracking as deeply invasive, lacking clear personal benefit, and contributing to a wider logic of human optimization. Some participants described this logic as psychologically disempowering or as a potential loss of control (75). As a result, some participants suggested age restrictions for EEG-supported CHTs.

Compared to academic discourse, which often focus on issues like managing incidental findings, technical safety, or employer monitoring [see e.g., (76–78)], participants’ concerns were more grounded in everyday social risks. In particular, they feared misuse by health insurers, or negative impacts on self-perception caused by inaccurate or poorly validated feedback. This is in line with studies that found scepticism about the necessity and reliability of EEG-supported CHTs in consumer contexts (79). Similar to sleep tracking technologies, which have been shown to inadvertently worsen sleep quality [e.g., (80)], EEG-based feedback was seen as potentially misleading, reinforcing anxiety rather than enhancing performance. This suggests a partial mismatch between user concerns and the risk scenarios most often highlighted in academic literature.

Overall, participants expressed a cautiously open attitude towards EEG-supported wearables, but identified multiple conditions for acceptability, including medical relevance, voluntariness, and transparent use. While existing studies often focus on specific applications such as emotion analysis [cf (81–83)] or therapeutic use in populations with attention disorders [cf (84)], comparative studies on the broader societal reception of EEG-supported CHTs remain limited. Our findings suggest that acceptance cannot be assumed based on clinical benefit alone, but must be evaluated in relation to how these devices affect autonomy, identity, and the implicit norms of human optimization in everyday contexts. Participants' repeated distinction between “step count” and “brain data” suggests that EEG signals are not merely experienced as another form of bodily data, but as something closer to the cognitive self. This aligns with neuroethical concepts such as mental privacy and cognitive liberty, which have gained prominence in recent years [e.g., (85–87)]. These concepts emphasize that brain data, by virtue of its perceived closeness to thought, intention, and affect may require special ethical treatment, including stricter protection against misuse or coercion. Drawing on participants' concerns, normative boundaries for the use of EEG-supported CHTs may include guarantees of voluntary use, strict limitations on data access (especially by insurers or employers), and clear prohibitions against persuasive or manipulative system feedback. Such guardrails could help prevent overreach in everyday environments, where cognitive technologies are not yet subject to robust governance.

These reflections underscore that autonomy is not merely practical concern, but a central ethical dimension in the evaluation of EEG-supported CHTs. While our study does not frame these technologies explicitly within the field of Neuroethics, the questions raised, particularly regarding agency, informed use, and optimization pressures, clearly resonate with current debates in that domain.

## 5 Conclusion

This study provides essential insights into how different types of users perceive data privacy and engage with CHTs, highlighting a range of perspectives, concerns, and reservations.

Our findings underline the importance of privacy to users, but show that most participants either read privacy information superficially or not at all. This behavior often reflects trust in large companies, as well as frustration with the inaccessible and complex nature of privacy documents. These observations highlight a persistent problem: privacy information is often not user-friendly, a concern that has been raised in previous studies [see e.g., (44)]. In addition, the study reveals that participants were generally unfamiliar with specific consent models prior to the study. Nevertheless, their preferences in the survey were clear: while some users accepted a Broad consent approach, the majority expressed a preference for Dynamic consent models, where research purposes and data recipients are clearly defined, or for Study-specific consent, allowing them to authorize data use on a case-by-case basis.

The study also suggests that CHTs may foster a greater focus on personal health goals among users who are already inclined towards health-conscious behaviors. While some participants felt that tracking their health data increased their sense of responsibility for maintaining health-promoting behaviors, opinions were divided, particularly regarding the perceived pressure or benefit of such tracking mechanisms. This trend aligns with findings indicating that individuals who are already health-conscious are more receptive to CHT-induced behavioral changes than the general population, signaling the importance of balanced design approaches for CHTs. Future research could explore how CHTs might be designed to support positive behavior change sustainably, without imposing excessive pressure or stress on users and without excluding or disadvantaging certain groups.

In this context, future studies should also focus more explicitly on the perspectives of digitally marginalized or health-vulnerable populations. While our study was based on a comparatively privileged and tech-savvy sample, there is a pressing need to understand how individuals with limited digital access, chronic illness, or lower socioeconomic status engage with, or are excluded from, the use and governance of CHTs. Purposeful sampling strategies and inclusive research designs will be essential to ensure that ethical frameworks are responsive to a broader spectrum of lived experiences.

Examining attitudes towards EEG-based technologies, another key aspect of our research, revealed mixed reactions. On the one hand, there is a degree of curiosity and openness, especially if such technologies can improve health or productivity. On the other hand, many participants expressed significant concerns about potential surveillance, unintended behavioral changes and long-term effects on mental health. These ambivalent views highlight a central tension between the potential benefits and ethical concerns associated with such technology. The findings suggest that users are cautious about the widespread adoption of EEG-supported CHTs and recognize that current designs may need significant refinement. But, research into EEG-supported CHTs and their use in everyday life is still in its infancy, and there are many unanswered questions that future studies should address. For example, it is unclear how these technologies can be designed to maximize user benefit while protecting user autonomy and privacy. Further research is needed to understand the long-term effects of these technologies on user behavior and mental health. In addition, ethical guidelines should be developed to regulate the use of EEG-supported CHTs in different areas of life. Here, existing guidelines, such as the *Recommendation on the Ethics of Neurotechnology*^10^, should also be considered.

In summary, this study shows that there are significant differences in users' privacy priorities, attitudes toward health accountability incentives, and ethical perspectives on advanced technologies like EEG-supported CHTs. With the increasing integration of CHTs into daily life, both in leisure and formal healthcare settings, and the rising demand for citizen-generated health data for research and commercial purposes, it is crucial to consider this diversity when developing future CHTs and privacy strategies. Future research, incorporating inter- but especially transdisciplinary approaches alongside active citizen involvement, should focus on better addressing individual privacy needs, supporting users' sense of health responsibility, and optimizing the benefits of CHTs and emerging technologies, such as EEG-supported CHTs in a digitalized world. In addition, the ethical governance of EEG-supported technologies should not be developed in expert circles alone. Participatory and deliberative approaches, involving a broad range of stakeholders, including users, healthcare providers, ethicists, and vulnerable groups, can play a key role in co-creating socially robust frameworks for responsible innovation. Such formats are particularly valuable for technologies that touch on cognitive autonomy and identity, where public values and experiential knowledge must be taken seriously from the outset. Ensuring equitable accessibility, not only in terms of device availability, but also in terms of comprehensibility, consent literacy, and contextual fit, will be just as vital as safeguarding autonomy. Only then can these technologies serve diverse populations fairly and meaningfully.

## 6 Limitations

It should be noted that this study is subject to methodological limitations, which may have an impact on the validity and generalizability of the findings. A significant challenge is the self-selection bias, whereby participants may have chosen to engage in the study due to their own interests or attitudes, which could result in a systematic sample bias and, consequently, raise questions about the representativeness of the results. Furthermore, there is a risk of social desirability influencing responses, as participants may provide answers that are perceived as socially acceptable or expected, rather than expressing their true beliefs. Furthermore, the relatively modest sample size—albeit typical for interview studies—constrains the statistical robustness of the findings and limits the ability to draw comprehensive and generalisable conclusions. It is also important to note that the study was predominantly composed of technology-enthusiast participants, a factor that, while expected for a study that is specifically focused on technologies and CHT use, may nonetheless shape the study's outcomes in ways that may not fully reflect broader public attitudes. In particular, the limited inclusion of digitally marginalized or health-vulnerable populations restricts the transferability of our findings to groups whose experiences with CHT, and their ethical evaluation, may differ significantly. Future research should therefore address this limitation through purposive sampling and inclusive design strategies.

## Data Availability

The raw data supporting the conclusions of this article will be made available by the authors, without undue reservation.

## Footnotes

^1^The term consumer health technologies (CHT), which we use in this paper, refers to wearable devices or health, lifestyle and fitness apps for smartphones that collect and analyse health and disease-related data such as heart rate, physical activity, mood or blood glucose, to name just a few of the most commonly collected parameters.

^2^The Open Humans platform is dedicated to empowering individuals and communities to explore and share their personal data for education, health and research purposes. Open Humans aims to help people access and understand their personal data and support them in doing and sharing things that utilise this data (see https://www.openhumans.org/about/).

^3^Smart4Health enables citizens to manage and bridge their own health data across the EU and beyond to promote personal and societal health and well-being. The Smart4Health research consortium, funded by the EU's Horizon 2020 framework programme, is developing a mobile app for this purpose that enables users to manage, share and donate their health data across the EU (see https://smart4health.eu/de/).

^4^This policy contrasts with approaches in many other countries, where public health coverage rarely extends to CHTs.

^5^Switzerland, for instance, has updated its Human Research Law in 2024, to reflect evolving technological and societal changes. (see for more details: https://www.swissmedic.ch/swissmedic/en/home/news/mitteilungen/neues-verordnungsrecht-ab-1-nov-2024.html

^6^MAXQDA is a proven, commercial tool from VERBI for computer-aided qualitative data and text analysis.

^7^The participant ID consists: cht following the participant number_Gender_Age; X=the respondents did not provide any information about their age.

^8^ https://www.generalivitality.com/about-our-program/

^9^While participants primarily expressed concerns about keeping brain activity data private and not sharing it with third parties such as health insurers, they generally did not articulate specific worries about other potential uses of these data or about how they could be further interpreted or analyzed. This difference may reflect a general gap between user perspectives and academic discussions on data privacy risks. Moreover, it is possible that the absence of detailed concerns could be due to the nature of the questions asked; a more in-depth inquiry into why participants felt certain boundaries were being crossed might have elicited further insights.

^10^ https://unesdoc.unesco.org/ark:/48223/pf0000391444
